# The course of children's mental health symptoms during and beyond the COVID-19 pandemic

**DOI:** 10.1017/S0033291724001491

**Published:** 2024-09

**Authors:** Joanne L. Park, Brae Anne McArthur, André Plamondon, Jackson M.A. Hewitt, Nicole Racine, Sheila McDonald, Suzanne Tough, Sheri Madigan

**Affiliations:** 1 Department of Psychology, University of Calgary, Calgary, AB, Canada; 2 Department of Psychology, Mount Royal University, Calgary, AB, Canada; 3 Alberta Children's Hospital Research Institute, Calgary, AB, Canada; 4 Département des fondements et pratiques en éducation, Université Laval, QC, Canada; 5 School of Psychology, Faculty of Social Sciences, University of Ottawa, Ottawa, ON, Canada; 6 Children's Hospital of Eastern Ontario Research Institute, Ottawa, ON, Canada; 7 Department of Pediatrics, Cumming School of Medicine, University of Calgary, Calgary, AB, Canada; 8 Department of Community Health Sciences, Cumming School of Medicine, University of Calgary, Calgary, AB, Canada

**Keywords:** child and adolescent, COVID-19 pandemic, longitudinal, mental health symptoms, post-pandemic, risk and resilience

## Abstract

**Background:**

The COVID-19 pandemic is associated with increases in child mental health problems, but the persistence of these changes in the post-pandemic era remains uncertain. Additionally, it is unclear whether changes in mental health problems during the pandemic exceed the anticipated increases as children age. This study controls for the linear effect of age in 1399 children, investigating the course of child-reported anxiety, depression, hyperactivity, and inattention symptoms during and after the pandemic, and identifies risk and protective factors that predict these mental health trajectories.

**Methods:**

Children (51% male; ages 9–11 at the first timepoint) provided mental health ratings at three pandemic timepoints (July–August 2020; March–April 2021; November 2021–January 2022) and one post-pandemic timepoint (January–July 2023). Mothers reported pre-pandemic mental health (2017–2019) and socio-demographic factors. Children reported socio-demographic factors, risk (e.g. screen time, sleep), and resilience (e.g. optimism) factors during the first timepoint.

**Results:**

Average mental health symptoms increased over time, with more children exceeding clinical cut-offs for poor mental health at each subsequent pandemic timepoint. Growth curve modeling, adjusting for age-related effects, revealed a curvilinear course of mental health symptoms across all domains. Examination of risk and protective factors revealed that pre-existing mental health symptoms and optimism were associated with the course of symptoms.

**Conclusions:**

After considering age effects, children's mental health follows a curvilinear pattern over time, suggesting an initial decline followed by a rising trend in symptoms post-COVID. These findings underscore the continued need for additional resources and timely, evidence-based mental health prevention and intervention for children.

## Introduction

An extensive body of literature highlights a sustained increase in mental health challenges among children during the pandemic, particularly among adolescents and girls (Kauhanen et al., 2023; Madigan et al., 2024; Racine et al., 2021), aligning with the significant disruptions experienced by children and families during this time, such as school closures, financial disruptions, and social isolation (Kerr, Rasmussen, Fanning, & Braaten, 2021; Viner et al., 2022). However, few studies have examined changes in children's mental health across more than two time points during the pandemic (Breaux et al., 2021; Essler, Christner, & Paulus, 2023) and none to our knowledge have examined changes post-COVID. Without this data to capture the evolving dynamics of child mental health in the context of the pandemic, it remains unknown whether the pandemic has had an enduring impact on children's mental health symptoms. It is crucial to ascertain this information to guide future research, policy, and practice initiatives.

Another challenge of the existing pandemic child mental health data lies in the complexity of disentangling pandemic effects from developmental effects. Specifically, studies have not distinguished whether the observed increases in mental health problems due to the pandemic are above typical developmental increases observed in children as they age (McLaughlin & King, 2015). This conflation can obfuscate the interpretation of the data. Consequently, a significant research gap remains in providing a comprehensive view of the trajectories of child mental health throughout and, more critically, after the pandemic, while also considering typical developmental patterns.

Existing pandemic mental health literature has predominantly focused on children's internalizing symptoms (Madigan et al., 2023b). Comparatively, examinations of longitudinal changes in symptoms of hyperactivity and inattention are sparse. Studying fluctuations of hyperactivity and inattention is important given recent evidence that stress due to the pandemic may manifest as externalizing problems (Breaux et al., 2021; Mohler-Kuo, Dzemaili, Foster, Werlen, & Walitza, 2021). Existing studies tend to demonstrate an increase in symptoms of inattention and hyperactivity during compared to before the pandemic (Rogers & MacLean, 2023). While previous research sheds light on the more immediate impacts of the pandemic on inattention and hyperactivity, there is a notable gap in our understanding of how these symptoms developed over the course of, and beyond, the pandemic.

### Risk and resilience factors

As outlined by the developmental psychopathology framework, child mental health is shaped by a complex interplay of individual and environmental factors (Cicchetti & Cohen, 1995; Rutter & Sroufe, 2000). Resilience theory further posits that specific qualities within the individual or their environment may aid in navigating adversity and maintaining positive mental health (Masten, 2001). Thus, it is essential to investigate factors previously linked to psychopathology in children as potential risk or protective elements influencing mental health trends throughout the pandemic (Masten & Motti-Stefanidi, 2020). Recent research on mental health during the pandemic has revealed a number of potential risk factors, including caregiver mental health (Gruhn et al., 2023), child sex, age, income levels, ethnicity (Madigan et al., 2023b; Shoshani, 2023), and screen time (Madigan et al., 2022). Conversely, resilience factors such as adaptive coping strategies or cognitions (e.g. optimism; Finch, Waters, and Farrell, 2023), quality of family relationships (McArthur, Racine, McDonald, Tough, & Madigan, 2023), and daily lifestyle routines such as physical activity and sleep (Neville et al., 2022; Richter, Ferraz-Rodrigues, Schilling, Camargo, & Nunes, 2023) have been identified. Optimism is important to examine due to its role in facilitating adaptive problem-solving strategies in the face of uncontrollable adversity such as a global pandemic (Mesman, Vreeker, & Hillegers, 2021), and relationships with parents and caregivers may be magnified during the pandemic when peers and other social connections were limited due to social distancing policies. Given the increased focus on describing the enduring effects of the pandemic on child mental health (Bhutta et al., 2022), identifying unique predictors of the course of mental health challenges is crucial for informing further policy actions, resource allocation, and recovery efforts aimed at addressing and restoring mental health post-COVID.

### The current study

The current study responds to editorial calls for more longitudinal follow-ups of existing cohorts to continuously assess the impact of the COVID-19 pandemic, particularly in a developmental context, and to identify risk and resilience factors (Cortese, Sabe, & Solmi, 2022; Sonuga-Barke & Fearon, 2021; Wade, Prime, & Browne, 2020). The first objective of the current study was to describe the trajectories of child-reported depression, anxiety, inattention, and hyperactivity symptoms across three timepoints during, and one timepoint following, the pandemic. We hypothesized that increases in children's symptoms would be observed over the course of the pandemic and, based on cumulative risk models (Evans, Li, & Whipple, 2013), would be sustained in the post-pandemic period. Second, in these analyses, we control for the linear effect of age to determine if any changes are over and above expected age-related increases in symptoms. While controlling for age at a single time point accounts for age-related differences, this approach allows us to distinguish changes in mental health symptoms during and post-pandemic from the natural progression of mental health symptoms that occur as children get older. We hypothesized that despite adjusting for age-related increases in symptoms, there would be increases in children's symptoms over the course of the pandemic, albeit to a lesser degree. Third, we examine risk and protective factors associated with symptom levels at pandemic onset and symptom change during and post-pandemic. Consistent with previous research, we hypothesized that pre-pandemic mental health, as well as socio-demographic, family, and individual risk factors would predict increases in symptoms, whereas protective factors would be associated with decreases in symptoms and recovery in the post-pandemic period.

## Method

### Participants

Children (*n* = 1399) were drawn from the All Our Families (AOF) cohort. Data that support the findings of this study are available from the All Our Families Cohort (https://ucalgary.ca/allourfamilies), and metadata of variables can be found at https://www.maelstrom-research.org/study/aof. Restrictions apply to the availability of these deidentified data, which were used according to data sharing agreements in Calgary, Canada (Table 1; McDonald et al., 2013; Tough et al., 2017). Women were recruited in pregnancy (*n* = 3387; August 2008–December 2010) and have been followed at 4 months, 1, 2, 3, 5, 8, 9, 10, 11, and 12 years postpartum. Inclusion criteria were: (1) >18 years, (2) fluent in English, (3) gestational age <24 weeks, and (4) receiving community-based prenatal care. Mothers who remained eligible (i.e. agreed to be contacted for additional research; *n* = 2445) were asked to participate in each of the three COVID-19 surveys and the post-COVID survey. For the first time in our cohort's history, they were asked to invite their children to participate, resulting in varying numbers of mothers and children participating at each timepoint: 1333 mothers (May–July, 2020) and 893 children (July–August, 2020) at COVID-1; 1361 mothers and 1045 children at COVID-2 (March–April, 2021); 1280 mothers and 1034 children at COVID-3 (November 2021–January, 2022); and 1529 mothers and 1317 children at the post-COVID timepoint (January–July, 2023). This timepoint is considered a post-COVID timepoint as it falls after the lifting of all mandatory public health restrictions in Alberta in June, 2022. Moreover, it corresponds with the World Health Organization's decision to downgrade the COVID-19 pandemic from a global health emergency to an ongoing health issue on May 5, 2023. In the regional area where this study took place, schools were closed from March 17, 2020, to September 1, 2020, and then remained open.

**Table 1.** Descriptive statistics and participant characteristics, *N* = 1399

Characteristic	% Missing	*N* (%)	M	s.d.	Min–Max
*Demographics*					
Age					
COVID-1 age, years^a^	42.6		10.29	0.78	9.06–11.89
COVID-2 age, years^a^	32.5		10.91	0.78	9.68–12.53
COVID-3 age, years^a^	32.9		11.63	0.76	10.41–13.25
Post-COVID age, years^a^	13.7		12.85	0.79	11.56–14.62
Sex assigned at birth^b^	0				
Female		686 (49.0)			
Male		713 (51.0)			
Racial/ethnic minority group^a^	16.0				
Yes		267 (19.1)			
No		908 (64.9)			
Self-identified race and ethnicity^a^	16.0				
Black		14 (1.0)			
East Asian		67 (4.8)			
Indigenous		15 (1.1)			
Latin American		19 (1.4)			
Middle Eastern		19 (1.4)			
South Asian		37 (2.6)			
Southeast Asian		25 (1.8)			
White		908 (64.9)			
Other		71 (5.1)			
COVID-1 household family income^b^	26.9				
<$ 80 000 per year		156 (11.2)			
$ 80 000^+^ per year		867 (62.0)			
*Pre-pandemic mental health symptoms*^b^					
Depression	7.0		50.23	9.72	37–103
Clinically significant depression		59 (4.2)			
Anxiety	6.9		49.42	10.20	28–94
Clinically significant anxiety		58 (4.1)			
Hyperactivity	6.9		50.19	9.72	34–100
Clinically significant hyperactivity		54(3.9)			
Inattention	6.7		49.63	9.30	35–80
Clinically significant inattention		24(1.7)			
*Predictor variables at COVID-1*					
Sleep, hrs per night^a^	42.7		9.23	0.89	6.5–10
Screen time, hrs per week^a^	43.0		23.59	11.94	0–49
Physical activity, days per week^a^	42.9		5.23	1.95	0–7
Connection to caregivers^a^	42.9		10.69	1.54	3–12
Optimism^a^	43.0		12.55	2.56	3–15
Maternal depression^b^	26.5		8.02	5.73	0–29

*Notes:* ^a^Reported by child; ^b^reported my mother.

A validity check item was incorporated into each survey, prompting children to reflect on the accuracy of their responses. The item read, ‘Sometimes it is hard to tell other people how you feel or what you think. My answers to this survey were:’, with response options ranging from 1 (*Not at all true*) to 4 (*Very much true*). Amongst children who provided assent and participated at any time-point (*n* = 1523), children who indicated ‘not at all true’ for at least one survey time-point (*n* = 124, 8.1%) reported fewer mental health symptoms across time-points (online Supplementary Table S8, available online), and therefore were considered distinct and were excluded from analysis. All other children with valid data for at least one timepoint were included in the analyses (*n* = 1399). All procedures were approved by the institutional Research Ethics Board (REB13-0868).

### Measures

#### Mental health

Child-reported mental health during and after COVID-19 was derived by creating a composite, standardized T-score (0–100 with a mean of 50 based on age norms) for *depression*, *anxiety*, *inattention*, and *hyperactivity* symptoms, respectively, on the Behavior Assessment System for Children (BASC-3; Reynolds and Kamphaus, 2015). A binary measure was derived indicating those who reached the ‘clinically significant’ range (T-score = 70^+^) for each respective mental health domain. Pre-pandemic mental health (2017–2019) was reported by mothers when children were age 8 (BASC-2; Reynolds and Kamphaus, 2004). Internal consistency was high across the study waves for both mother-reported and self-reported scores (*α* = 0.81 to *α* = 0.93).

#### Risk and protective factors

Demographic characteristics included *child biological sex* (reported by mothers; male v. female), *age* at each timepoint, *ethnicity* (reported by child at the Post-COVID timepoint; majority population v. historically minoritized), and *total household income* prior to COVID-19 (⩾80 000 v. <80 000 $ CAD). The following factors were reported by children at COVID-1. *Screen time:* time spent using electronic devices on a typical weekday and weekend day outside of schoolwork (0 = *none*; to 7 = *7 or more hours*; a weighted weekly average was derived). *Physical activity*: number of days in the last week children participated in physical activity that made their heart rate go up for at least 60 min (0 = *0 days* to 7 = *7 days*). *Sleep*: number of hours children slept on a typical night (1 = *less than 7* *h* to 8 = *10* *h or more*). *Connectedness to parents*: measured via 3-items on the Middle Years Development Instrument (MDI; Schonert-Reichl et al., 2013; α = 0.70). *Optimism*: also measured using 3-items on the MDI (α = 0.77). At COVID-1, mothers reported on *maternal depression* in the past two-weeks using the 10-item Center for Epidemiological Studies of Depression Short Form (Radloff, 1977; α = 0.87).

### Statistical analysis

**Figure 1.**
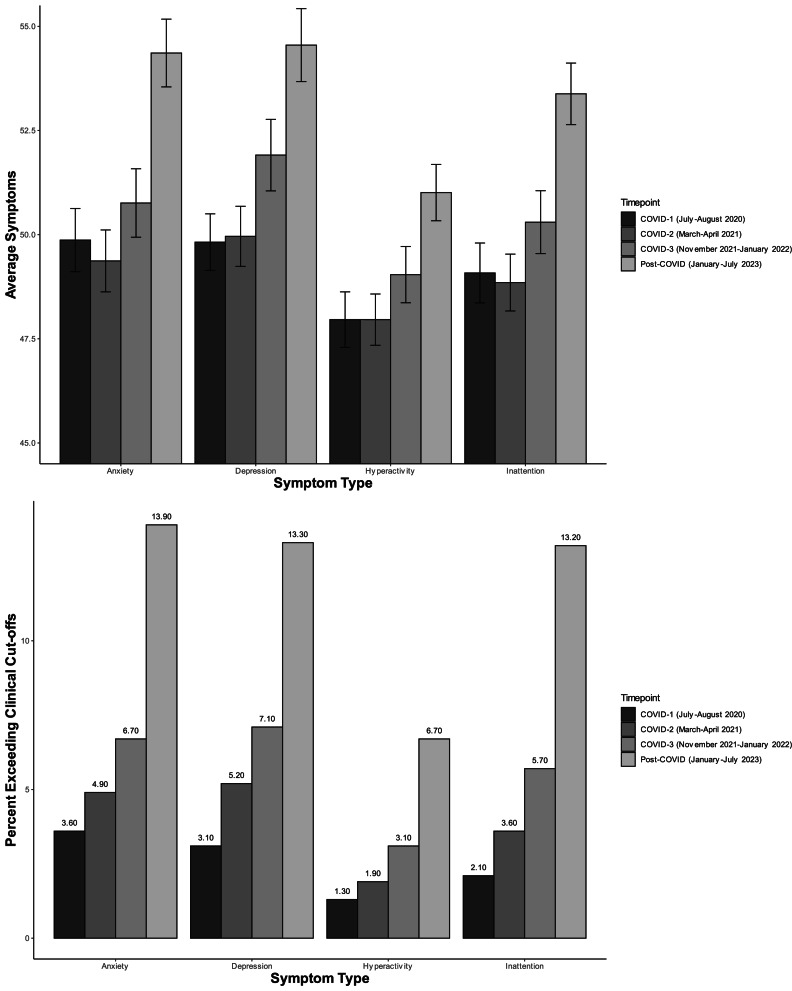
Mean mental health symptom scores (top) and percent of children exceeding clinically significant cut-offs (bottom) from the first COVID-19 timepoint (COVID-1) to the post-COVID-19 timepoint (post-COVID).

Changes in levels of child mental health symptoms and the proportion of children with clinically significant levels of symptoms were examined graphically (Fig. 1) and with means and frequencies from the onset of the COVID-19 pandemic compared to later COVID-19 timepoints and the post-COVID-19 timepoint.

Using MPlus 8.0 (Muthén & Muthén, 2017), we used a multilevel modeling framework to estimate growth curve models of child mental health symptoms across four timepoints. Multilevel growth models combine fixed effects (average population parameter) and random effects (variability around parameters) to capture inter-individual variability in intra-individual patterns of change (Curran, Obeidat, & Losardo, 2010). We used a sequential model selection approach to identify the best fitting trajectory of each symptom type across the pandemic. We first compared a baseline random intercept-only model (null model) to a linear model with a random slope to capture individual variability in the rate of linear change. Last, we examined a quadratic model with random slopes. All models except the null model were specified while controlling for age at each time point (as a within-person variable) as a linear fixed effect, to capture the trajectory of mental health symptoms over and above the effect of age. Model comparisons were performed via likelihood ratio tests using the MplusAutomation package (Hallquist & Wiley, 2018) in RStudio (Posit Team, 2023). For all outcomes, the quadratic model was found to be the best-fitting model (see online Supplementary Tables S1–S4, available online). Once the best-fitting model was selected, risk and protective predictors of the intercepts and slopes were added. Full information maximum likelihood estimation with the MLR estimator was used to account for potential non-normality and the presence of missing data (Enders, 2001).

## Results

### Descriptive characteristics

Descriptive characteristics are presented in Table 1. Mean depression and anxiety were significantly higher at the COVID-3 and post-COVID surveys when compared to the COVID-1 survey (see Fig. 1 and Table 2). Mean hyperactivity and inattention were only significantly higher at the post-COVID survey when compared to the COVID-1 survey. The proportion of children meeting the clinically significant cut-off (T-score 70+) for each of the mental health domains were significantly higher at COVID-2, COVID-3, and post-COVID compared to COVID-1.

**Table 2.** Youth self-reported mental health symptoms across the COVID-19 pandemic

Characteristic	Clinical cut-off *N* (%)	M	s.d.
*Depression symptoms*			
COVID-1	44 (3.1)	49.82	9.80
COVID-2	73 (5.2)^a^	49.96	11.26
COVID-3	99 (7.1)^a^	51.91^b^	13.34
Post-COVID	186 (13.3)^a^	54.55^b^	15.36
*Anxiety symptoms*			
COVID-1	50 (3.6)	49.87	10.92
COVID-2	69 (4.9)^a^	49.37	11.61
COVID-3	94 (6.7)^a^	50.76^b^	12.77
Post-COVID	194 (13.9)^a^	54.36^b^	14.28
*Hyperactivity symptoms*			
COVID-1	18 (1.3)	47.96	9.61
COVID-2	26 (1.9)^a^	47.96	9.61
COVID-3	43(3.1)^a^	49.04	10.50
Post-COVID	94 (6.7)^a^	51.01^b^	11.87
*Inattention symptoms*			
COVID-1	30 (2.1)	49.08	10.37
COVID-2	51 (3.6)^a^	48.85	10.66
COVID-3	80 (5.7)^a^	50.30	11.74
Post-COVID	185 (13.2)^a^	53.38^b^	12.97

a Frequency differs significantly from COVID-1 frequency score, using a Bonferroni adjusted alpha level of *p* < 0.004.

b Mean differs significantly from COVID-1 mean symptom score, using a Bonferroni adjusted alpha level of *p* < 0.004.

### Mental health trends across the COVID-19 pandemic

**Figure 2.**
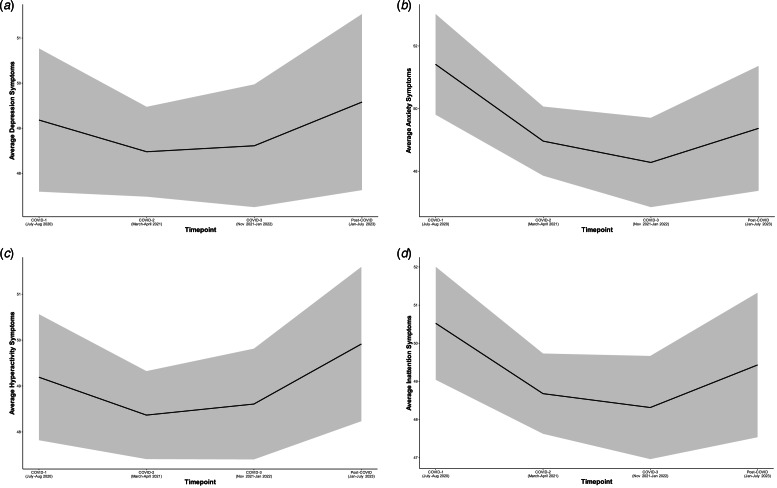
Model-estimated trajectories of child mental health symptoms from COVID-1 to post-COVID timepoints for depression (Panel A), anxiety (Panel B), hyperactivity (Panel C), and inattention (Panel D). Grey shaded area represents the 95% confidence bands.

Results from all growth models while controlling for age are presented in online Supplementary Tables S1-S4 (available online). Model-estimated trajectories of child depressive, anxiety, hyperactivity, and inattention symptoms, respectively, across time from the best-fitting model are presented in Fig. 2. For each mental health variable, change over time is best captured by the quadratic model (Model 3), after controlling for the linear effect of age. Model-derived estimates for each mental health symptom type for each timepoint are compared in online Supplementary Supplement 1 (available online).

The model for depression is characterized by no initial linear change on average (*b* = −1.12), followed by an average accelerated increase in the rate of change of symptoms throughout the pandemic and post-pandemic timepoints, as indicated by a significant quadratic slope (*b* = 0.42). This demonstrates a curvilinear pattern with relatively higher symptoms at the post-COVID timepoint compared to symptoms at COVID-2 and COVID-3 (Fig. 2a).

For anxiety, hyperactivity, and inattention, the models reveal an initial linear decrease in symptoms on average (*b* = −3.34; *b* = −1.36; *b* = −2.58, respectively), followed by an accelerated increase in the rate of change of symptoms (*b* = 0.89; *b* = 0.53; *b* = 0.74, respectively), resulting in a U-shaped curvilinear trend in symptoms (Figs 2b, 2c, and 2d). Initial symptom levels (intercepts) for all symptom types are not associated with the average rate of linear or quadratic change. However, higher rates of linear change are associated with smaller quadratic change for symptoms of depression (*b* = −21.24), anxiety (*b* = −16.90), and hyperactivity (*b* = −8.31), with a similar, but non-significant trend for inattention (*b* = −7.93).

### Socio-demographic predictors

Predictors of mental health symptoms were then included in the models (Table 3).

**Table 3.** Predictors of child mental health symptoms at COVID-19 pandemic onset and change over time (*N* = 1399)

	Depressive symptoms		Anxiety symptoms		Hyperactivity symptoms		Inattention symptoms	
	Estimate	95% CI	Estimate	95% CI	Estimate	95% CI	Estimate	95% CI
*Fixed effects*								
Age	0.44	(−0.10, 0.98)	−0.32	(−0.94, 0.29)	−0.36	(−0.92, 0.19)	0.30	(−0.30, 0.90)
Intercept	**49.65**	**(48.14, 51.16)**	**52.34**	**(50.84, 53.84)**	**49.25**	**(47.91, 50.58)**	**50.70**	**(49.31, 52.09)**
Linear slope	−1.03	(−2.44, 0.37)	**−3.24**	**(−4.52, −1.97)**	**−1.35**	**(−2.39, −0.31)**	**−2.61**	**(−3.75, −1.47)**
Quadratic slope	**0.47**	**(0.18, 0.77)**	**1.01**	**(0.75, 1.28)**	**0.53**	**(0.31, 0.75)**	**0.75**	**(0.51, 1.00)**
*Random effects*								
Intercept	33.98	(−56.09, 124.04)	43.75	(−16.58, 104.07)	**69.33**	**(26.60, 112.06)**	24.82	(−26.13, 75.77)
Linear slope	**110.03**	**(26.07, 193.98)**	**75.50**	**(21.32, 129.69)**	**36.11**	**(0.65, 71.56)**	26.17	(−18.62, 70.97)
Quadratic slope	**5.36**	**(1.97, 8.74)**	**3.82**	**(1.62, 6.03)**	**1.83**	**(0.43, 3.23)**	1.76	(−0.01, 3.52)
*Symptoms at COVID-19 onset (Intercept)*								
Pre-COVID symptoms	0.08	(−1.51, 1.67)	0.13	(−0.00, 0.26)	**0.33**	**(0.19, 0.46)**	**0.57**	**(0.42, 0.71)**
Racial/ethnic minority status	−0.82	(−4.46, 2.82)	−1.53	(−5.12, 2.06)	−1.76	(−4.91, 1.39)	−0.44	(−3.72, 2.85)
Income <$ 80 000	−0.03	(−4.49, 4.43)	−0.10	(−4.13, 3.93)	−1.31	(−4.81, 2.19)	2.05	(−1.39, 5.48)
Sex (male)	−0.92	(−3.78, 1.94)	1.23	(−1.49, 3.95)	−1.39	(−3.80, 1.03)	−1.96	(−4.54, 0.62)
Sleep	−0.15	(−2.28, 1.97)	−1.93	(−3.97, 0.11)	−1.15	(−2.81, 0.51)	−0.77	(−2.56, 1.02)
Screen time	0.38	(−1.30, 2.06)	0.07	(−0.06, 0.19)	0.07	(−0.04, 0.18)	0.07	(−0.04, 0.19)
Physical activity	0.01	(−0.95, 0.97)	0.11	(−0.78, 0.98)	0.02	(−0.70. 0.74)	0.11	(−0.65, 0.88)
Optimism	**−2.17**	**(−3.01, −1.34)**	**−3.04**	**(−3.74, −2.34)**	−0.49	(−1.16, 0.17)	−0.55	(−1.23, 0.14)
Maternal depression	−0.62	(−2.33, 1.10)	−0.05	(−0.32, 0.23)	−0.14	(−0.37, 0.08)	−0.13	(−0.37, 0.12)
Connection to caregivers	**−1.82**	**(−3.09, −0.55)**	−0.16	(−1.32, 1.00)	−0.01	(−1.02, 1.00)	−0.84	(−1.91, 0.23)
*Linear slope from COVID-1 to Post-COVID*								
Pre-COVID symptoms	**1.83**	**(0.22, 3.45)**	**0.16**	**(0.04, 0.28)**	0.12	(−0.00, 0.23)	−0.07	(−0.20, 0.06)
Racial/ethnic minority status	−0.91	(−4.39, 2.57)	0.79	(−2.44, 4.03)	1.51	(−1.10, 4.12)	0.79	(−2.03, 3.61)
Income <$ 80 000	1.12	(−3.29, 5.52)	0.79	(−3.04, 4.63)	1.27	(−1.74, 4.28)	−2.16	(−5.26, 0.93)
Sex (male)	0.67	(−2.15, 3.48)	−0.10	(−2.66, 2.45)	0.10	(−1.99, 2.19)	−0.57	(−2.90, 1.76)
Sleep	0.31	(−1.89, 2.51)	1.37	(−0.68, 3.42)	1.38	(−0.07, 2.83)	0.37	(−1.32, 2.06)
Screen time	0.34	(−1.45, 2.12)	0.00	(−0.12, 0.13)	0.03	(−0.08, 0.13)	0.02	(−0.09, 0.14)
Physical activity	0.22	(−0.78, 1.21)	−0.20	(−1.10, 0.69)	0.17	(−0.48, 0.82)	−0.29	(−1.02, 0.43)
Optimism	0.50	(−0.33, 1.32)	**1.22**	**(0.56, 1.88)**	−0.07	(−0.67, 0.52)	−0.23	(−0.86, 0.40)
Maternal depression	0.77	(−1.00, 2.53)	0.11	(−0.15, 0.36)	0.14	(−0.05, 0.34)	0.12	(−0.12, 0.35)
Connection to caregivers	0.76	(−0.54, 2.06)	0.26	(−0.83, 1.35)	−0.05	(−0.97, 0.88)	0.36	(−0.65, 1.37)
*Quadratic slope from COVID-1 to post-COVID*								
Pre-COVID symptoms	−0.31	(−0.62, 0.01)	**−0.03**	**(−0.06 −0.01)**	**−0.03**	**(−0.05, −0.01)**	0.01	(−0.02, 0.03)
Racial/ethnic minority status	0.30	(−0.38, 0.98)	−0.23	(−0.86, 0.41)	−0.37	(−0.87, 0.14)	−0.26	(−0.80, 0.29)
Income <$ 80 000	−0.38	(−1.23, 0.48)	−0.26	(−1.03, 0.51)	−0.35	(−0.95, 0.24)	0.40	(−0.24, 1.03)
Sex (male)	0.30	(−0.26, 0.86)	0.45	(−0.06, 0.96)	0.17	(−0.24, 0.59)	0.44	(−0.02, 0.90)
Sleep	−0.14	(−0.59, 0.32)	−0.24	(−0.66, 0.18)	−0.29	(−0.58, 0.01)	−0.01	(−0.35, 0.33)
Screen time	−0.09	(−0.45, 0.28)	−0.00	(−0.03, 0.03)	−0.01	(−0.03, 0.01)	−0.01	(−0.03, 0.02)
Physical activity	−0.06	(−0.25, 0.14)	0.06	(−0.12, 0.24)	−0.04	(−0.17, 0.10)	0.07	(−0.08, 0.21)
Optimism	−0.07	(−0.24, 0.09)	**−0.21**	**(−0.35, −0.07)**	0.01	(−0.11, 0.13)	0.04	(−0.09, 0.17)
Maternal depression	−0.09	(−0.44, 0.26)	−0.02	(−0.07, 0.03)	−0.02	(−0.06, 0.01)	−0.01	(−0.06, 0.03)
Connection to caregivers	−0.11	(−0.38, 0.15)	−0.07	(−0.29, 0.15)	0.01	(−0.18, 0.19)	−0.08	(−0.29, 0.13)

*Note*: Variables in bold are statistically significant at *p* < 0.05.

#### Depression

Greater optimism and higher perceived connection to caregivers predicted lower levels of initial depressive symptoms. Pre-pandemic depression symptoms were associated with the linear slope. None of the socio-demographic predictors were significantly associated with the quadratic growth of depressive symptoms over time.

#### Anxiety

Only greater optimism predicted lower levels of initial anxiety symptoms. Pre-pandemic anxiety symptoms and optimism at the start of the pandemic were both predictors of the linear slope and quadratic slope. Examination of online Supplementary Figure S1 (available online) suggests that, initially (COVID-1), all children showed similarly high levels of anxiety, but those with lower pre-pandemic anxiety demonstrated a relative decrease in anxiety over time with a slight increase at the end, while those with higher levels of pre-pandemic anxiety maintained relatively stable higher levels of anxiety across the pandemic. Examination of online Supplementary Figure S2 (available online) reveals that optimism primarily differentiated children at COVID-1. Over time, the slopes converged, such that all children became more similar in levels of anxiety.

#### Hyperactivity

Predictors of higher hyperactivity scores at pandemic-onset (intercept) included higher pre-pandemic hyperactivity scores. Only pre-pandemic hyperactivity was a predictor of the quadratic slope. Examination of online Supplementary Figure S3 (available online) indicates that children with higher levels of pre-pandemic hyperactivity demonstrated relatively stable, higher levels of hyperactivity through the pandemic, while those with lower levels of pre-pandemic hyperactivity demonstrated relatively lower levels of hyperactivity symptoms over time.

#### Inattention

Predictors of higher inattentive scores at pandemic-onset (intercept) included only higher inattention scores pre-pandemic. There were no significant predictors of the quadratic slope.

## Discussion

This study provides insights into the course of child mental health symptoms across four time points from early to post-COVID-19 pandemic. Descriptive results indicated an overall decline in the mental health of children as the pandemic unfolded, with an increasing number exceeding clinical cut-offs for depression, anxiety, hyperactivity, and inattention symptoms. However, growth curve modeling adjusting for age-related developmental effects showed that the course of each type of mental health symptoms followed a curvilinear trend - starting at a relatively higher level at pandemic onset (COVID-1, during school closures), exhibiting a decline (i.e. improvement) across COVID-2 and COVID-3, and then rising during the post-COVID period back to initial levels (COVID-1). Additionally, this study identified key risk and resiliency factors as predictors of the initial levels and course of mental health symptoms.

When examining the average levels of psychopathology symptoms during and post-COVID-19 (Fig. 1), our findings revealed that children reported higher mean rates of symptoms at COVID-3 (for depression and anxiety) and the post-COVID timepoint (all mental health symptoms) compared to the early stages of the pandemic (COVID-1). Consistent with these observations, the post-COVID assessment revealed a substantial rise in the percentage of children surpassing clinically significant cut-offs for depression (3–13%), anxiety (4–14%), inattention (2–13%) and hyperactivity (1–7%).

Growth curve models, which controlled for the fixed linear developmental effect of age, offer contextual insights into these increases. Contrary to the steep increases in mental health symptoms observed in Fig. 1, our findings suggest that the overall rise in mental health symptoms due to the pandemic may be less pronounced, suggesting that age-related factors seem to account for some of the increases in symptoms in Fig. 1. Despite this, the significant linear or quadratic slopes across all mental health symptoms indicates that the observed changes in mental health problems cannot be solely attributed to developmental effects, wherein there are rising incidences of mental health symptoms during emerging adolescence (Costello, Copeland, & Angold, 2011). Examination of the growth curve trajectories (Fig. 2) suggests potential trends in children's mental health symptoms, starting at average levels at COVID-1, decreasing during COVID-2 and COVID-3, and then demonstrating an increasing pattern at the post-COVID timepoint. It is important to clarify that these observations describe the curvilinear trend and do not indicate model-estimated statistical differences between specific waves.

Interpreting these patterns in the context of local public health restrictions, the slightly higher levels of anxiety and inattention symptoms at COVID-1 (compared to COVID-2 and COVID-3) may be associated with the social isolation, months of school closures and educational loss, limited access to extracurricular activities, and the diminished access to mental health supports during the early part of the pandemic. This trend aligns with findings from other studies, which demonstrated increased emergency department mental health visits for children during school closures compared to when schools were reopened (Newton et al., 2023), and greater mental health symptoms when learning online compared to in-person (Tsujimoto et al., 2023). The elevated symptoms during this timepoint compared to later timepoints may have been associated with these substantive changes to daily life routines (Hawrilenko, Kroshus, Tandon, & Christakis, 2021; Viner et al., 2022).

The pattern of decreasing symptoms in COVID-2 and COVID-3 seen in the growth curve models may be attributed to various factors, such as the return to school (September 2020) serving as a coping resource for students or a potential adjustment to the changes and stressors brought about by the pandemic. However, the unexpected rising trend in symptoms at the post-COVID timepoint, even after controlling for the developmental effect of age, and despite the conclusion of widespread pandemic public health restrictions and a perceived ‘return to normal,’ raises concerns. It is possible that the relatively elevated symptom levels and the increases in children meeting clinical cutoffs for mental health symptomatology at the post-COVID timepoint may be linked, in part, to an accumulation of stressors and their impact over time (Korczak, Madigan, & Vaillancourt, 2022; Sonuga-Barke & Fearon, 2021; Wade et al., 2020). This observation aligns with models of cumulative risk (Evans et al., 2013), suggesting that as the pandemic and its related stressors accumulated, an increasing number of children experienced a decline in their mental health. Importantly, this pattern of decline persists following the conclusion of the pandemic, highlighting the enduring impact of these stressors.

It is also possible that the initial decreases in mental health symptoms during COVID-2 and COVID-3, after adjusting for the linear effect of age, could also be attributed to lowered expectations and increased accommodations for students during this period. Examples include reduced schooling demands, exam cancellations, and time-limited initiatives aimed at supporting students (Minkos & Gelbar, 2021). However, the more recent upward trend may be linked to the reinstatement of ‘normal’ expectations and the cessation of these time-limited supports for students. Indeed, studies have highlighted the inadequacy of resources for children both prior to the pandemic (Waddell, McEwan, Shepherd, Offord, & Hua, 2005) and throughout its duration (Madigan et al., 2023a; Saunders et al., 2022). This deficiency in support is likely to have persisted into the post-pandemic period, contributing to the observed trends in mental health symptoms.

The examination of risk and resiliency factors revealed additional insights into the course of children's mental health during and beyond the pandemic. Pre-existing hyperactivity and inattention symptoms emerged as predictors of initial levels of these symptoms during the pandemic. This finding aligns with research highlighting the stability of these symptoms from childhood into early adolescence (Larsson, Larsson, & Lichtenstein, 2004). In contrast, pre-existing anxiety and depression symptoms predicted a steeper linear slope in their respective trajectories over time, suggesting that these internalizing problems may be more sensitive to the impact of stressors.

We found that heightened pre-pandemic anxiety and hyperactivity symptoms predicted their respective quadratic slopes. Online Supplementary Figure S1 illustrates that on average, children—regardless of pre-pandemic anxiety levels—experienced relatively higher anxiety at the first COVID timepoint, possibly reflecting the significant stress and uncertainty during that period. Those with higher pre-pandemic anxiety symptoms continued to experience higher levels of anxiety across time, while those with lower pre-pandemic anxiety experienced less anxiety at the second and third timepoints, with some elevations observed at the post-pandemic timepoint. This finding is consistent with two types of sensitization effects: (1) that individuals with pre-pandemic anxiety may be ‘sensitized’ to the additional stressors due to the pandemic, and (2) the pandemic may be a ‘sensitizing’ event for those with less pre-pandemic anxiety increasing vulnerability for later stress (Madigan et al., 2023b; Wade et al., 2020). The increased anxiety experienced by both groups at the post-pandemic timepoint may also reflect the impact of cumulative stress becoming evident after some time, or a delayed effect due to the removal of temporary adaptations and supports provided to students during the pandemic, as mentioned previously.

Examination of online Supplementary Figure S3 suggests a different interpretation of hyperactivity symptoms. Children with higher levels of pre-pandemic hyperactivity consistently displayed elevated levels throughout the pandemic. In contrast, those with lower pre-pandemic hyperactivity demonstrated relatively less symptoms that followed a shallow curvilinear pattern. This finding implies that, unlike internalizing symptoms such as anxiety, stable genetic effects may play a more influential role in hyperactivity symptoms compared to situational effects, such as the stress induced by the pandemic (Larsson et al., 2004).

In terms of resiliency factors, optimism was associated with lower initial levels of internalizing symptomatology and the quadratic slope of anxiety symptoms over time. Examination of online Supplementary Figure S2 suggests that the most robust differentiating effect of optimism occurred during the first timepoint. Over time, the impact of optimism assessed at COVID-1 appears to diminish, as illustrated by the converging pattern of slopes. This effect of optimism, at least regarding initial levels of symptoms, aligns with studies indicating that optimism both predicts positive mental health outcomes, and buffers against stressors (Rincón Uribe, Neira Espejo, & Pedroso, 2022). These findings support efforts to foster and cultivate optimistic attitudes on an ongoing basis within children as an important bulwark against poor mental health outcomes during stressful situations, and especially against anxiety.

Lastly, our study also found that children who reported feeling a strong connection to caregivers had lower levels of self-reported depression symptoms at the onset of the COVID-19 pandemic. However, connection to caregivers did not predict symptom trends across the pandemic time points. It is important to note that this measure primarily gauged the presence of a supportive adult in the child's home and therefore may not adequately capture other aspects of the caregiver-child relationship that have been identified as important predictors of child mental health over time (e.g. family conflict; Essler, Christner, and Paulus, 2021; Gruhn et al., 2023).

We did not find that minority status, income, child sex, sleep, screen time, physical activity, or maternal depression predicted the course of mental health symptoms, contrary to our expectations. Given that our sample largely comprised families with higher income and belonging to the majority group in Canada, the effects of these variables may have been underestimated. Furthermore, the inclusion of numerous covariates in our models may have reduced the ability to detect unique effects. Online Supplementary Tables S5-S7 illustrate bivariate correlations among risk and resilience factors, and their associations with child mental health symptoms across timepoints, showing that, at least at the bivariate level, associations align with expectations.

## Limitations

Several study limitations should be noted. First, the age range of children in our sample restricts the generalizability beyond middle childhood and early adolescence. Second, the socio-demographic characteristics of our sample are primarily representative of the geographical location of study participants, which includes higher parent educational attainment and family income. As such, this may have underestimated the impact of variables such as ethnicity and income and may limit the general applicability of our findings to rural and less educated and lower income urban areas. Third, our study results may primarily apply to children who experienced school closures solely during the initial phase of the pandemic. Results should be validated in other jurisdictions that encountered more intermittent or varied school closure policies or formats. Fourth, while we utilized standardized questionnaire instruments commonly employed in clinical practice, these may not match the diagnostic precision achievable through clinical interviews or a cross-informant approach. Some of our measures included a limited number of items to reduce participant burden for filling out multiple surveys. Additionally, including objective measures of covariates such as screen time, sleep time, or physical activity, may enhance the accuracy and reliability of these measures. Lastly, our study revealed substantial unexplained variability within the observed trend of quadratic slope increases over time. Thus, it is likely that unmeasured variables contributed to the diverse trajectories of mental health during and after the pandemic. Our study lacked a stress or adversity measure, which may be important to include in future research.

## Conclusions

While our results initially suggested a significant deterioration in child mental health across and after the pandemic, we found that these changes were somewhat mitigated when age-related increases in mental health difficulties were considered. Children appeared to exhibit an improvement midway through the pandemic; however, a subsequent rising trend in symptoms occurred during the post-pandemic period. As such, there is a pressing need for widespread access to timely and evidence-based mental health prevention and intervention to support children's well-being, as children experience lingering mental health challenges. Furthermore, our results emphasize the need for ongoing longitudinal monitoring to discern the developmental impacts, including cumulative and sensitization effects, of the COVID-19 pandemic on child mental health.

## Supporting information

Park et al. supplementary material 1 Park et al. supplementary material

Park et al. supplementary material 2 Park et al. supplementary material

Park et al. supplementary material 3 Park et al. supplementary material

Park et al. supplementary material 4 Park et al. supplementary material

## Supplementary material

The supplementary material for this article can be found at https://doi.org/10.1017/S0033291724001491.

## References

[ref1] Bhutta, Z. A., Boerma, T., Black, M. M., Victora, C. G., Kruk, M. E., & Black, R. E. (2022). Optimising child and adolescent health and development in the post-pandemic world. The Lancet, 399(10337), 1759–1761. 10.1016/S0140-6736(21)02789-6 35489362

[ref2] Breaux, R., Dvorsky, M. R., Marsh, N. P., Green, C. D., Cash, A. R., Shroff, D. M., … Becker, S. P. (2021). Prospective impact of COVID-19 on mental health functioning in adolescents with and without ADHD: Protective role of emotion regulation abilities. Journal of Child Psychology and Psychiatry and Allied Disciplines, 62(9), 1132–1139. 10.1111/jcpp.13382 33543486 PMC8014657

[ref3] Cicchetti, D., & Cohen, D. J. (1995). Perspectives on developmental psychopathology. In D. Cicchetti, & D. J. Cohen (Eds.), Developmental psychopathology, Vol. 1: Theory and methods (pp. 3–20). New York: John Wiley & Sons.

[ref4] Cortese, S., Sabe, M., & Solmi, M. (2022). Editorial perspective: COVID-19-related publications on young people's mental health – what have been the key trends so far and what should come next? Journal of Child Psychology and Psychiatry and Allied Disciplines, 63, 1671–1673. 10.1111/jcpp.13615 35438193 PMC9114924

[ref5] Costello, E. J., Copeland, W., & Angold, A. (2011). Trends in psychopathology across the adolescent years: What changes when children become adolescents, and when adolescents become adults? Journal of Child Psychology and Psychiatry and Allied Disciplines, 52(10), 1015–1025. 10.1111/j.1469-7610.2011.02446.x 21815892 PMC3204367

[ref6] Curran, P. J., Obeidat, K., & Losardo, D. (2010). Twelve frequently asked questions about growth curve modeling. Journal of Cognition and Development, 11(2), 121–136. 10.1080/15248371003699969 21743795 PMC3131138

[ref7] Enders, C. K. (2001). The impact of nonnormality on full information maximum-likelihood estimation for structural equation models with missing data. Psychological Methods, 6, 352–370. 10.1037/1082-989x.6.4.352 11778677

[ref8] Essler, S., Christner, N., & Paulus, M. (2021). Longitudinal relations between parental strain, parent–child relationship quality, and child well-being during the unfolding COVID-19 pandemic. Child Psychiatry and Human Development, 52(6), 995–1011. 10.1007/s10578-021-01232-4 34426893 PMC8382101

[ref9] Essler, S., Christner, N., & Paulus, M. (2023). Short-term and long-term effects of the COVID-19 pandemic on child psychological well-being: A four-wave longitudinal study. *European Child and Adolescent Psychiatry*, 33, 909–922. 10.1007/s00787-023-02215-7 37119393 PMC10148581

[ref10] Evans, G. W., Li, D., & Whipple, S. S. (2013). Cumulative risk and child development. Psychological Bulletin, 139(6), 1342–1396. 10.1037/a0031808 23566018

[ref11] Finch, J., Waters, A. M., & Farrell, L. J. (2023). Adolescent anxiety, depression and flourishing before and during COVID-19 and the predictive role of baseline psychological capital (PsyCap) on student mental health and subjective wellbeing during the pandemic. Child Psychiatry and Human Development. Advance online publication. 10.1007/s10578-023-01568-z PMC11928354 37418072

[ref12] Gruhn, M., Miller, A. B., Machlin, L., Motton, S., Thinzar, C. E., & Sheridan, M. A. (2023). Child anxiety and depression symptom trajectories and predictors over 15 months of the coronavirus pandemic. Research on Child and Adolescent Psychopathology, 51(2), 233–246. 10.1007/s10802-022-00963-9 36048373 PMC9435416

[ref13] Hallquist, M. N., & Wiley, J. F. (2018). MplusAutomation: An R package for facilitating large-scale latent variable analyses in M plus. Structural Equation Modeling: A Multidisciplinary Journal, 25(4), 621–638. 10.1080/10705511.2017.1402334 30083048 PMC6075832

[ref14] Hawrilenko, M., Kroshus, E., Tandon, P., & Christakis, D. (2021). The association between school closures and child mental health during COVID-19. JAMA Network Open, 4(9), 1–11. 10.1001/jamanetworkopen.2021.24092 PMC8417763 34477850

[ref15] Kauhanen, L., Wan Mohd Yunus, W. M. A., Lempinen, L., Peltonen, K., Gyllenberg, D., Mishina, K., … Sourander, A. (2023). A systematic review of the mental health changes of children and young people before and during the COVID-19 pandemic. European Child and Adolescent Psychiatry, 32(6), 995–1013. 10.1007/s00787-022-02060-0 35962147 PMC9373888

[ref16] Kerr, M. L., Rasmussen, H. F., Fanning, K. A., & Braaten, S. M. (2021). Parenting during COVID-19: A study of parents’ experiences across gender and income levels. Family Relations, 70(5), 1327–1342. 10.1111/fare.12571 34548726 PMC8444790

[ref17] Korczak, D. J., Madigan, S., & Vaillancourt, T. (2022). Data Divide - Disentangling the role of the COVID-19 pandemic on child mental health and well-being. JAMA Pediatrics, 176(7), 635–636. 10.1002/ab.21986 35467721

[ref18] Larsson, J. O., Larsson, H., & Lichtenstein, P. (2004). Genetic and environmental contributions to stability and change of ADHD symptoms between 8 and 13 years of age: A longitudinal twin study. Journal of the American Academy of Child and Adolescent Psychiatry, 43(10), 1267–1275. 10.1097/01.chi.0000135622.05219.bf 15381894

[ref19] Madigan, S., Korczak, D. J., Vaillancourt, T., Racine, N., Hopkins, W. G., Pador, P., … Neville, R. D. (2023a). Comparison of paediatric emergency department visits for attempted suicide, self-harm, and suicidal ideation before and during the COVID-19 pandemic: A systematic review and meta-analysis. The Lancet Psychiatry, 10(5), 342–351. 10.1016/s2215-0366(23)00036-6 36907199 PMC10097509

[ref20] Madigan, S., Racine, N., Vaillancourt, T., Korczak, D. J., Hewitt, J. M. A., Pador, P., … Neville, R. D. (2023b). Changes in depression and anxiety among children and adolescents from before to during the COVID-19 pandemic: A systematic review and meta-analysis. JAMA Pediatrics, 177(6), 567–581. 10.1001/jamapediatrics.2023.0846 37126337 PMC10152379

[ref21] Madigan, S., Vaillancourt, T., Dimitropoulos, G., Premji, S., Kahlert, S. M., Zumwalt, K., … Neville, R. D. (2024). A systematic review and meta-analysis: Child and adolescent healthcare utilization for eating disorders during the COVID-19 pandemic. Journal of the American Academy of Child and Adolescent Psychiatry. Advance online publication. 10.1016/j.jaac.2024.02.009 38431196

[ref22] Masten, A. S. (2001). Ordinary magic: Resilience processes in development. American Psychologist, 56(3), 227–238. 10.1037/0003-066X.56.3.227 11315249

[ref23] Masten, A. S., & Motti-Stefanidi, F. (2020). Multisystem resilience for children and youth in disaster: Reflections in the context of COVID-19. Adversity and Resilience Science, 1(2), 95–106. 10.1007/s42844-020-00010-w 32838305 PMC7314620

[ref24a] Madigan, S., Eirich, R., Pador, P., McArthur, B. A., & Neville, R. D. (2022). Assessment of changes in child and adolescent screen time during the COVID-19 pandemic. JAMA Pediatrics, 176, 1188–1198. 10.1001/jamapediatrics.2022.4116 36342702 PMC9641597

[ref24] McArthur, B. A., Racine, N., McDonald, S., Tough, S., & Madigan, S. (2023). Child and family factors associated with child mental health and well-being during COVID-19. European Child and Adolescent Psychiatry, 32(2), 223–233. 10.1007/s00787-021-01849-9 34302530 PMC8302979

[ref25] McDonald, S. W., Lyon, A. W., Benzies, K. M., Mcneil, D. A., Lye, S. J., Dolan, S. M., … Tough, S. C. (2013). The All Our Babies pregnancy cohort: design, methods, and participant characteristics. 10.1186/1471-2393-13-S1-S2 PMC3561154 23445747

[ref26] McLaughlin, K. A., & King, K. (2015). Developmental trajectories of anxiety and depression in early adolescence. Journal of Abnormal Child Psychology, 43(2), 311–323. 10.1007/s10802-014-9898-1 24996791 PMC4286282

[ref27] Mesman, E., Vreeker, A., & Hillegers, M. (2021). Resilience and mental health in children and adolescents: An update of the recent literature and future directions. Current Opinion in Psychiatry, 34(6), 586–592. 10.1097/YCO.0000000000000741 34433193 PMC8500371

[ref28] Minkos, M. L., & Gelbar, N. W. (2021). Considerations for educators in supporting student learning in the midst of COVID-19. Psychology in the Schools, 58(2), 416–426. 10.1002/pits.22454 33362299 PMC7753346

[ref29] Mohler-Kuo, M., Dzemaili, S., Foster, S., Werlen, L., & Walitza, S. (2021). Stress and mental health among children/adolescents, their parents, and young adults during the first COVID-19 lockdown in Switzerland. International Journal of Environmental Research and Public Health, 18(9), 4668. 10.3390/ijerph18094668 33925743 PMC8124779

[ref30] Muthén, L. K., & Muthén, B. O. (2017). MPLus user's guide (8th ed.). Los Angeles, CA: Muthén & Muthén.

[ref31] Neville, R. D., Lakes, K. D., Hopkins, W. G., Tarantino, G., Draper, C. E., Beck, R., & Madigan, S. (2022). Global changes in child and adolescent physical activity during the COVID-19 pandemic: A systematic review and meta-analysis. JAMA Pediatrics, 176(9), 886–894. 10.1001/jamapediatrics.2022.2313 35816330 PMC9274449

[ref32] Newton, A. S., Xie, J., Wright, B., Lategan, C., Winston, K., & Freedman, S. B. (2023). Visits to Alberta emergency departments for child mental health concerns during the COVID-19 pandemic: An examination of visit trends in relation to school closures and reopenings. Pediatric Emergency Care, 39(7), 542–547. 10.1097/PEC.0000000000002979 37246141 PMC10317191

[ref33] Posit Team (2023). RSTudio: Integrated development environment for R. Boston, MA: Posit Software, PBC.

[ref34] Racine, N., McArthur, B. A., Cooke, J. E., Eirich, R., Zhu, J., & Madigan, S. (2021). Global prevalence of depressive and anxiety symptoms in children and adolescents during COVID-19: A meta-analysis. JAMA Pediatrics, 175(11), 1142–1150. 10.1001/jamapediatrics.2021.2482 34369987 PMC8353576

[ref35] Radloff, L. S. (1977). The CES-D scale: A self-report depression scale for research in the general population. Applied Psychological Measurement, 1(3), 385–401. 10.1177/014662167700100306

[ref36] Reynolds, C. R., & Kamphaus, R. W. (2004). The behavior assessment system for children (2nd ed.). Bloomington, MN: Pearson.

[ref37] Reynolds, C. R., & Kamphaus, R. W. (2015). Behavior assessment system for children, third edition *(*BASC-3*)*. Bloomington, MN: Pearson.

[ref38] Richter, S. A., Ferraz-Rodrigues, C., Schilling, L. B., Camargo, N. F., & Nunes, M. L. (2023). Effects of the COVID-19 pandemic on sleep quality in children and adolescents: A systematic review and meta-analysis. Journal of Sleep Research, 32(1), Article e13720. 10.1111/jsr.13720 36000251 PMC9539085

[ref39] Rincón Uribe, F. A., Neira Espejo, C. A., & Pedroso, J. da S. (2022). The role of optimism in adolescent mental health: A systematic review. Journal of Happiness Studies, 23, 815–845. 10.1007/s10902-021-00425-x

[ref40] Rogers, M. A., & MacLean, J. (2023). ADHD symptoms increased during the COVID-19 pandemic: A meta-analysis. Journal of Attention Disorders, 27(8), 800–811. 10.1177/10870547231158750 36879524 PMC9996113

[ref41] Rutter, M., & Sroufe, L. A. (2000). Developmental psychopathology: Concepts and challenges. Development and Psychopathology, 12(3), 265–269. 10.1017/s0954579400003023 11014739

[ref42] Saunders, N. R., Stukel, T. A., Strauss, R., Fu, L., Cohen, E., Guttmann, A., & Toulany, A. (2022). Changes in hospital-based care seeking for acute mental health concerns among children and adolescents during the COVID-19 pandemic in Ontario, Canada, through September 2021. JAMA Network Open, 5(7), e2220553. 10.1001/jamanetworkopen.2022.20553 35797049 PMC9264033

[ref43] Schonert-Reichl, K. A., Guhn, M., Gadermann, A. M., Hymel, S., Sweiss, L., & Hertzman, C. (2013). Development and validation of the Middle Years Development Instrument (MDI): Assessing children's well-being and assets across multiple contexts. Social Indicators Research, 114(2), 345–369. 10.1007/s11205-012-0149-y 24109151 PMC3790250

[ref44] Shoshani, A. (2023). Longitudinal changes in children's and adolescents’ mental health and well-being and associated protective factors during the COVID-19 pandemic. Psychological Trauma: Theory, Research, Practice, and Policy. Advance online publication. 10.1037/tra0001556 37498719

[ref45] Sonuga-Barke, E., & Fearon, P. (2021). Editorial: Do lockdowns scar? Three putative mechanisms through which COVID-19 mitigation policies could cause long-term harm to young people's mental health. Journal of Child Psychology and Psychiatry and Allied Disciplines, 62(12), 1375–1378. 10.1111/jcpp.13537 34806768 PMC9011706

[ref46] Tough, S. C., Mcdonald, S. W., Collisson, B. A., Graham, S. A., Kehler, H., Kingston, D., & Benzies, K. (2017). Cohort profile: The All Our Babies pregnancy cohort (AOB). International Journal of Epidemiology, 46, 1389–1390. 10.1093/ije/dyw363 28180262

[ref47] Tsujimoto, K. C., Cost, K. T., LaForge-MacKenzie, K., Anagnostou, E., Birken, C. S., Charach, A., … Korczak, D. J. (2023). School and learning contexts during the COVID-19 pandemic: Implications for child and youth mental health. Current Psychology, 42(34), 29969–29985. 10.1007/s12144-022-03941-y PMC9685153 36468159

[ref48] Viner, R., Russell, S., Saulle, R., Croker, H., Stansfield, C., Packer, J., … Minozzi, S. (2022). School closures during social lockdown and mental health, health behaviors, and well-being among children and adolescents during the first COVID-19 wave: A systematic review. JAMA Pediatrics, 176(4), 400–409. 10.1001/jamapediatrics.2021.5840 35040870

[ref49] Waddell, C., McEwan, K., Shepherd, C. A., Offord, D. R., & Hua, J. M. (2005). A public health strategy to improve the mental health of Canadian children. Canadian Journal of Psychiatry, 50(4), 226–233. 10.1177/070674370505000406 15898462

[ref50] Wade, M., Prime, H., & Browne, D. T. (2020). Why we need longitudinal mental health research with children and youth during (and after) the COVID-19 pandemic. Psychiatry Research, 290(May), 113143. 10.1016/j.psychres.2020.113143 32502829 PMC7253952

