# Supplementary material for: The course of children's mental health symptoms during and beyond the COVID-19 pandemic
Source: Psychol Med. 2024 Sep 9;54(12):3345–56. doi: 10.1017/S0033291724001491 (PMC11496214; doi:10.1017/S0033291724001491)
Supplement: Park et al. supplementary material 1 — Park et al. supplementary material [file S0033291724001491sup001.docx]

Figure S1. Average anxiety trajectories of individuals with average pre-pandemic anxiety, low pre-pandemic anxiety (1SD below the mean) and high pre-pandemic (1 SD above the mean). Grey shaded area represents the 95% Confidence Bands.

**
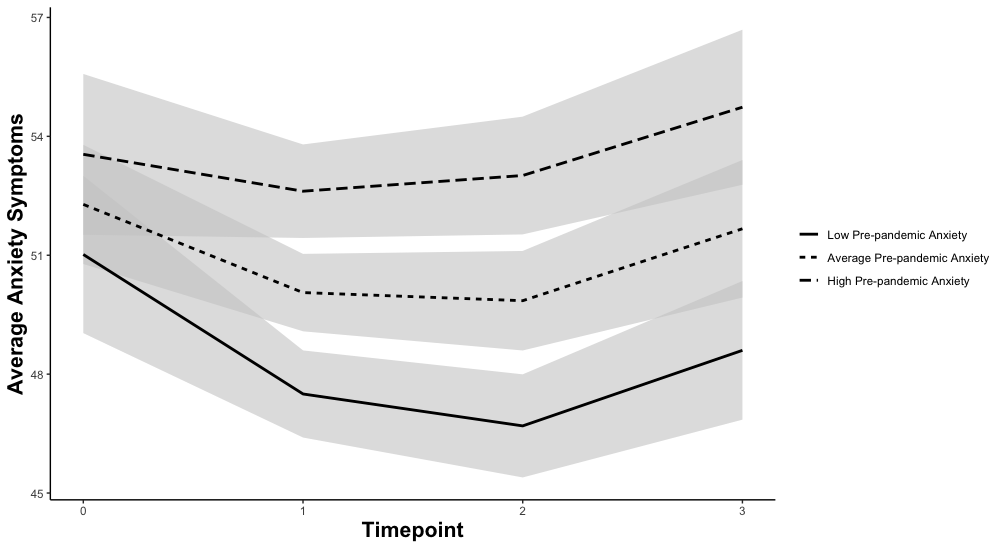
**
