# Supplementary material for: The course of children's mental health symptoms during and beyond the COVID-19 pandemic
Source: Psychol Med. 2024 Sep 9;54(12):3345–56. doi: 10.1017/S0033291724001491 (PMC11496214; doi:10.1017/S0033291724001491)
Supplement: Park et al. supplementary material 2 — Park et al. supplementary material [file S0033291724001491sup002.docx]

Figure S2. Average anxiety trajectories of individuals experiencing average levels of optimism, low optimism (1SD below the mean) and high optimism (1 SD above the mean). Grey shaded area represents the 95% Confidence Bands.

**
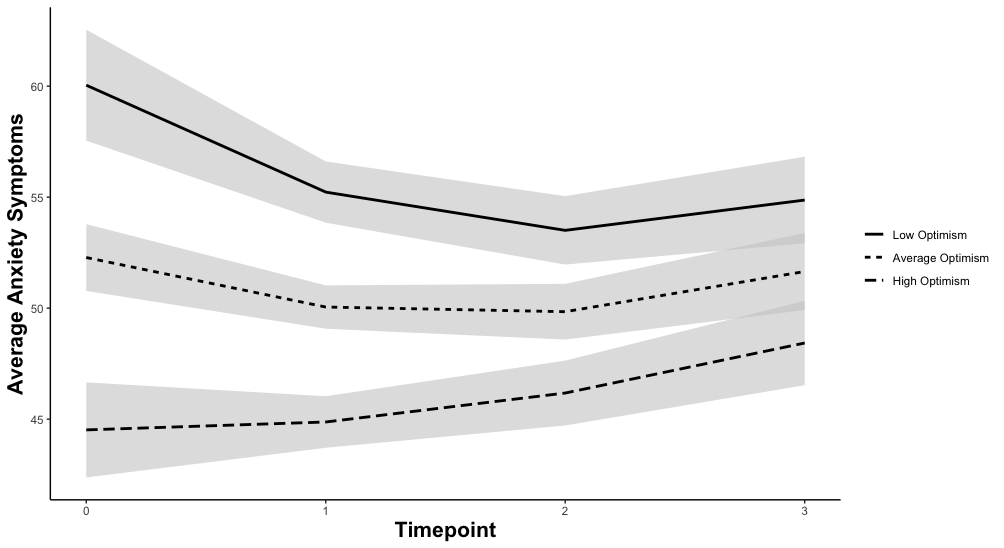
**
