# Supplementary material for: The course of children's mental health symptoms during and beyond the COVID-19 pandemic
Source: Psychol Med. 2024 Sep 9;54(12):3345–56. doi: 10.1017/S0033291724001491 (PMC11496214; doi:10.1017/S0033291724001491)
Supplement: Park et al. supplementary material 4 — Park et al. supplementary material [file S0033291724001491sup004.docx]

**Table S1.**

Fixed effects, variance (random) components, and fit statistics for sequential model testing for symptoms of depression.

|  | **Model 1:**  Null | **Model 2:** Random linear slope, controlling for age | **Model 3:** Random linear and quadratic slopes, controlling for age | **Unconditional Model 3:** Random linear and quadratic slopes |
| --- | --- | --- | --- | --- |
|  | Estimate (*SE*) | Estimate (*SE*) | Estimate (*SE*) | Estimate (*SE*) |
| **Means** |  |  |  |  |
| Intercept | **51.96 (0.30)** | **46.87 (0.42)** | **49.18 (0.81)** | **50.04 (0.76)** |
| Linear slope |  | **0.66 (0.33)** | -1.12 (0.72) | -1.00 (0.72) |
| Quadratic slope |  |  | **0.42 (0.15)** | **0.54 (0.14)** |
| Age |  | **1.30 (0.34)** | **0.87(0.36)** |  |
|  |  |  |  |  |
| **Variances** |  |  |  |  |
| Intercept | **80.83 (6.00)** | **68.54 (15.17)** | 72.34 (50.22) | 71.60 (49.95) |
| Linear slope |  | **13.49 (1.90)** | **101.22 (43.75)** | **102.80 (43.52)** |
| Quadratic slope |  |  | **5.05 (1.75)** | **5.11 (1.74)** |
|  |  |  |  |  |
| **Covariances** |  |  |  |  |
| Intercept-Linear Slope |  | **-14.72 (4.79)** | -56.98 (44.37) | -57.31 (44.01) |
| Intercept-Quadratic Slope |  |  | 11.59 (8.45) | 11.67 (8.40) |
| Linear-Quadratic Slope |  |  | **-21.24 (8.63)** | **-21.52 (8.58)** |
|  |  |  |  |  |
| Log likelihood | -14963.10 | -14721.71 | -14682.26 | - 14695.33 |
| AIC | 29932.20 | 29457.41 | 29386.51 | 29410.66 |
| BIC | 29950.96 | 29501.19 | 29455.31 | 29473.22 |

Note. Significant parameters (*p*  < .05) are bolded. Intraclass correlation for the null model = 0.47

**Table S2.**

Fixed effects, variance (random) components, and fit statistics for sequential model testing for symptoms of anxiety.

|  | **Model 1:**  Null | **Model 2:** Random linear slope, controlling for age | **Model 3:** Random linear and quadratic slopes, controlling for age | **Unconditional Model 3:** Random linear and quadratic slopes |
| --- | --- | --- | --- | --- |
|  | Estimate (*SE*) | Estimate (*SE*) | Estimate (*SE*) | Estimate (*SE*) |
| **Means** |  |  |  |  |
| Intercept | **51.44 (0.30)** | **46.51 (0.44)** | **51.41 (0.82)** | **51.96 (0.75)** |
| Linear slope |  | 0.46 (0.32) | **-3.34 (0.67)** | **-3.25 (0.66)** |
| Quadratic slope |  |  | **0.89 (0.14)** | **0.96 (0.13)** |
| Age |  | **1.43 (0.35)** | 0.55 (0.37) |  |
|  |  |  |  |  |
| **Variances** |  |  |  |  |
| Intercept | **90.15 (4.96)** | **98.16 (11.34)** | **110.89 (33.85)** | **110.29 (33.75)** |
| Linear slope |  | **9.72 (1.21)** | **80.30 (28.77)** | **80.55 (28.68)** |
| Quadratic slope |  |  | **4.00 (1.17)** | **4.00 (1.17)** |
|  |  |  |  |  |
| **Covariances** |  |  |  |  |
| Intercept-Linear Slope |  | **-13.64 (3.30)** | -52.76 (29.21) | -52.67 (29.13) |
| Intercept-Quadratic Slope |  |  | 10.17 (5.58) | 10.16 (5.56) |
| Linear-Quadratic Slope |  |  | **-16.90 (5.71)** | **-16.94 (5.69)** |
|  |  |  |  |  |
| Log likelihood | -14712.54 | -14525.304 | -14479.41 | -14490.19 |
| AIC | 29431.08 | 29064.61 | 28980.82 | 29000.38 |
| BIC | 29449.85 | 29108.39 | 29049.61 | 29062.93 |

Note. Significant parameters (*p*  < .05) are bolded. Intraclass correlation for the null model = 0.55

**Table S3.**

Fixed effects, variance (random) components, and fit statistics for sequential model testing for symptoms of hyperactivity.

|  | **Model 1:**  Null | **Model 2:** Random linear slope, controlling for age | **Model 3:** Random linear and quadratic slopes, controlling for age | **Unconditional Model 3:** Random linear and quadratic slopes |
| --- | --- | --- | --- | --- |
|  | Estimate (*SE*) | Estimate (*SE*) | Estimate (*SE*) | Estimate (*SE*) |
| **Means** |  |  |  |  |
| Intercept | **49.28 (0.25)** | **46.19 (0.38)** | **49.19 (0.70)** | **48.85 (0.63)** |
| Linear slope |  | 0.86 (0.27) | **-1.36 (0.54)** | **-1.39 (0.54)** |
| Quadratic slope |  |  | **0.53 (0.11)** | **0.48 (0.11)** |
| Age |  | 0.28 (0.29) | -0.34 (0.31) |  |
|  |  |  |  |  |
| **Variances** |  |  |  |  |
| Intercept | **66.32 (3.70)** | **80.88 (7.62)** | **87.18 (23.42)** | **86.94 (23.34)** |
| Linear slope |  | **7.50 (0.92)** | **40.14 (18.52)** | **39.18 (18.40)** |
| Quadratic slope |  |  | **2.04 (0.73)** | **2.00 (0.72)** |
|  |  |  |  |  |
| **Covariances** |  |  |  |  |
| Intercept-Linear Slope |  | **-12.34 (2.37)** | -30.50 (19.99) | -29.66 (19.87) |
| Intercept-Quadratic Slope |  |  | 5.24 (3.79) | 5.04 (3.77) |
| Linear-Quadratic Slope |  |  | **-8.31 (3.61)** | **-8.12 (3.59)** |
|  |  |  |  |  |
| Log likelihood | -13958.01 | -13809.976 | -13782.085 | -13791.89 |
| AIC | 27922.03 | 27633.95 | 27586.17 | 27603.77 |
| BIC | 27940.80 | 27677.74 | 27654.97 | 27666.33 |

Note. Significant parameters (*p*  < .05) are bolded. Intraclass correlation for the null model = 0.58

**Table S4.**

Fixed effects, variance (random) components, and fit statistics for sequential model testing for symptoms of inattention.

|  | **Model 1:**  Null | **Model 2:** Random linear slope, controlling for age | **Model 3:** Random linear and quadratic slopes, controlling for age | **Unconditional Model 3:** Random linear and quadratic slopes |
| --- | --- | --- | --- | --- |
|  | Estimate (*SE*) | Estimate (*SE*) | Estimate (*SE*) | Estimate (*SE*) |
| **Means** |  |  |  |  |
| Intercept | **50.92 (0.28)** | **46.38 (0.41)** | **50.52 (0.76)** | **50.89 (0.68)** |
| Linear slope |  | 0.48 (0.30) | **-2.58 (0.59)** | **-2.53 (0.59)** |
| Quadratic slope |  |  | **0.74 (0.13)** | **0.79 (0.12)** |
| Age |  | **1.25 (0.33)** | 0.38 (0.35) |  |
|  |  |  |  |  |
| **Variances** |  |  |  |  |
| Intercept | **81.72 (3.76)** | **87.30 (8.47)** | **71.82 (27.44)** | **66.31 (26.81)** |
| Linear slope |  | **6.99 (0.96)** | 34.29 (23.66) | 29.75 (23.08) |
| Quadratic slope |  |  | **2.11 (0.93)** | **1.94 (0.91)** |
|  |  |  |  |  |
| **Covariances** |  |  |  |  |
| Intercept-Linear Slope |  | **-9.85 (2.58)** | -14.25 (24.58) | -9.38 (23.94) |
| Intercept-Quadratic Slope |  |  | 3.11 (4.70) | 2.20 (4.60) |
| Linear-Quadratic Slope |  |  | -7.93 (4.62) | -7.05 (4.53) |
|  |  |  |  |  |
| Log likelihood | -14336.521 | -14168.209 | -14130.52 | -14142.946 |
| AIC | 28679.04 | 28350.42 | 28283.04 | 28305.89 |
| BIC | 28697.81 | 28394.20 | 28351.84 | 28368.45 |

Note. Significant parameters (*p*  < .05) are bolded. Intraclass correlation for the null model = 0.58

**Table S5.**

|  | **1** | **2** | **3** | **4** | **5** | **6** | **7** | **8** | **9** | **10** |
| --- | --- | --- | --- | --- | --- | --- | --- | --- | --- | --- |
| Child Sex  (Male = 1; Female = 2) **(1)** | -- |  |  |  |  |  |  |  |  |  |
| Child Age at COVID-1 **(2)** | -.006 | -- |  |  |  |  |  |  |  |  |
| Ethnicity (0 = Majority; 1 = Minority) **(3)** | .004 | -.024 | -- |  |  |  |  |  |  |  |
| Income **(4)** | .017 | .012 | -.102*** | -- |  |  |  |  |  |  |
| Screentime **(5)** | -.056 | .073 | .083* | -.076* | -- |  |  |  |  |  |
| Physical activity **(6)** | -.066 | -.052 | -.079* | .103** | -.244*** | -- |  |  |  |  |
| Sleep **(7)** | -.045 | -.125*** | -.129*** | .122** | -.191*** | .139*** | -- |  |  |  |
| Connections with Caregivers **(8)** | .023 | -.015 | -.079* | .028 | -.137*** | .047 | .149*** | -- |  |  |
| Optimism **(9)** | -.013 | -.061 | -.038 | .022 | -.158*** | .127*** | .170*** | .437*** | -- |  |
| Maternal Depression **(10)** | -.033 | -.017 | .013 | -.121*** | .099** | -.093** | -.092** | -.056 | -.140*** | -- |

Bivariate correlations among risk and resilience factors.

Note. *p < .05; **p < .01; ***p < .001 Only child age at COVID-1 was included in this table for the sake of simplicity. Child sex was reported by mothers. Ethnicity was reported by children at the Post-COVID timepoint. Income was reported by mothers prior to COVID-19. Maternal depression was assessed at COVID 1. All other variables were assessed at COVID-1.

|  | **Depression** | | | | **Anxiety** | | | |
| --- | --- | --- | --- | --- | --- | --- | --- | --- |
|  | COVID-1 | COVID-2 | COVID-3 | Post-COVID | COVID-1 | COVID-2 | COVID-3 | Post-COVID |
| Child Sex (Male = 1; Female = 2) | -.023 | .071* | .106** | .213*** | .083* | .144*** | .169*** | .290*** |
| Child Age at COVID-1 | .021 | .112** | .149*** | .089* | .008 | .029 | .107** | .077* |
| Ethnicity (0 = Majority; 1 = Minority) | -.029 | -.032 | .003 | .015 | -.023 | -.025 | -.043 | -.059* |
| Income | -.055 | -.080* | -.114*** | -.020 | -.022 | -.018 | -.056 | .010 |
| Screentime | .158** | .150*** | .096* | .085* | .168*** | .163*** | .115** | .103** |
| Physical activity | -.055 | -.098* | -.064 | -.092* | -.113** | -.124** | -.081* | -.052 |
| Sleep | -.126*** | -.117** | -.138*** | -.156*** | -.176*** | -.096* | -.101* | -.088* |
| Connections with Caregivers | -.416*** | -.301*** | -.242*** | -.195*** | -.220*** | -.152*** | -.139*** | -.142*** |
| Optimism | -.591*** | -.461*** | -.360*** | -.316*** | -.539*** | -.380*** | -.328*** | -.307*** |
| Maternal Depression | .136*** | .114** | .167*** | .105** | .162*** | .139*** | .145*** | .084* |

**Table S6.**

Bivariate correlations between risk and resilience factors and depression and anxiety across time points.

Note. *p < .05; **p < .01; ***p < .001 Only child age at COVID-1 was included in this table for the sake of simplicity. Child sex was reported by mothers. Ethnicity was reported by children at the Post-COVID timepoint. Income was reported by mothers prior to COVID-19. Maternal depression was assessed at COVID 1. All other variables were assessed at COVID-1.

**Table S7.**

|  | **Hyperactivity** | | | | **Inattention** | | | |
| --- | --- | --- | --- | --- | --- | --- | --- | --- |
|  | COVID-1 | COVID-2 | COVID-3 | Post-COVID | COVID-1 | COVID-2 | COVID-3 | Post-COVID |
| Child Sex (Male = 1; Female = 2) | -.138*** | -.106** | -.093** | .019 | -.187*** | -.104** | .071* | .056* |
| Child Age at COVID-1 | -.105** | -.044 | .010 | .021 | -.038 | .032 | .057 | .073* |
| Ethnicity (0 = Majority; 1 = Minority) | -.050 | -.026 | -.033 | -.068* | .032 | .001 | .028 | -.035 |
| Income | -.017 | -.024 | -.022 | .004 | -.050 | -.049 | -.068* | -.044 |
| Screentime | .161*** | .173*** | .139*** | .069 | .203*** | .203*** | .177*** | .131*** |
| Physical activity | -.054 | -.061 | -.041 | -.039 | -.133*** | -.126** | -.129** | -.078* |
| Sleep | -.074* | -.050 | -.058 | -.062 | -.145*** | -.102* | -.114** | -.053 |
| Connections with Caregivers | -.148*** | -.134*** | -.171*** | -.107** | -.266*** | -.227*** | -.242*** | -.202*** |
| Optimism | -.213*** | -.235*** | -.213*** | -.184*** | -.314*** | -.316*** | -.297*** | -.261*** |
| Maternal Depression | .142*** | .140*** | .165*** | .083* | .166*** | .135*** | .194*** | .120*** |

Bivariate correlations between risk and resilience factors and hyperactivity and inattention across timepoints.

Note. *p < .05; **p < .01; ***p < .001 Only child age at COVID-1 was included in this table for the sake of simplicity. Child sex was reported by mothers. Ethnicity was reported by children at the Post-COVID timepoint. Income was reported by mothers prior to COVID-19. Maternal depression was assessed at COVID 1. All other variables were assessed at COVID-1.

**Table S8**

Comparison of mean mental health symptoms between included and excluded participants based on validity item responses

|  | **Included Participants**  **(n = 1399)** | **Excluded Participants**  **(n = 124)** | **Welch’s Test** |
| --- | --- | --- | --- |
|  | M (SD) | M (SD) | p-value |
| **COVID-1** |  |  |  |
| Depression | 49.82 (9.80) | 45.79 (6.81) | < .001 |
| Anxiety | 49.87 (10.92) | 45.06 (8.83) | < .001 |
| Hyperactivity | 47.96 (9.61) | 47.45 (9.46) | .637 |
| Inattention | 49.08 (10.37) | 49.20 (10.04) | .915 |
| **COVID-2** |  |  |  |
| Depression | 49.96 (11.26) | 44.71 (5.69) | < .001 |
| Anxiety | 49.37 (11.61) | 44.73 (9.72) | <.001 |
| Hyperactivity | 47.96 (9.61) | 48.38 (10.41) | .724 |
| Inattention | 48.85 (10.66) | 47.36 (10.48) | .218 |
| **COVID-3** |  |  |  |
| Depression | 51.91 (13.34) | 45.68 (7.94) | < .001 |
| Anxiety | 50.76 (12.77) | 44.85 (10.04) | <.001 |
| Hyperactivity | 49.04 (10.50) | 46.78 (10.48) | .048 |
| Inattention | 50.30 (11.74) | 47.04 (10.50) | .005 |
| **Post-COVID** |  |  |  |
| Depression | 54.55 (15.36) | 46.62 (10.46) | < .001 |
| Anxiety | 54.36 (14.28) | 46.03 (11.82) | < .001 |
| Hyperactivity | 51.01 (11.87) | 47.42 (11.26) | .002 |
| Inattention | 53.38 (12.97) | 48.67 (12.18) | < .001 |

**Appendix S1.**

|  | **Depression** | **Anxiety** | **Hyperactivity** | **Inattention** |
| --- | --- | --- | --- | --- |
|  | Mean (95% CI) | Mean (95% CI) | Mean (95% CI) | Mean (95% CI) |
| COVID-1 | 49.18 (47.59, 50.77) | 51.41 (49.81, 53.02) | 49.19 (47.82, 50.56) | 50.52 (49.04, 52.00) |
| COVID-2 | 48.48 (47.48, 49.47) | 48.96 (47.86, 50.07) | 48.37 (47.41, 49.32) | 48.68 (47.63, 49.73) |
| COVID-3 | 48.61 (47.25, 49.97) | 48.28 (46.86, 49.71) | 48.61 (47.40, 49.81) | 48.31 (46.96, 49.67) |
| Post-COVID | 49.58 (47.63, 51.53) | 49.37 (47.38, 51.36) | 49.91 (48.23, 51.59) | 49.43 (47.54, 51.32) |

**Table S9.** Model derived estimates from the model with random linear and quadratic slopes and controlling for age as a fixed effect for each time point with 95% Confidence Intervals.

When examining the model derived estimates for each mental health symptom type (Table S9) for each timepoint, only COVID-1 anxiety was significantly higher than all other timepoints. Additionally, COVID-1 inattention was significantly higher than symptoms at COVID-2 and COVID-3, but not the post-COVID timepoint. There were no significant differences among the model derived estimates for each of the timepoints for depression and hyperactivity symptoms.
